# Kinetics of COVID-19 mRNA primary and booster vaccine-associated neutralizing activity against SARS-CoV-2 variants of concern in long-term care facility residents: a prospective longitudinal study in Japan

**DOI:** 10.1186/s12979-023-00368-2

**Published:** 2023-08-17

**Authors:** Tomoyuki Kakugawa, Keiko Doi, Yuichi Ohteru, Hiroyuki Kakugawa, Keiji Oishi, Masahiro Kakugawa, Tsunahiko Hirano, Yusuke Mimura, Kazuto Matsunaga

**Affiliations:** 1 Department of Pulmonology and Gerontology, Graduate School of Medicine, Yamaguchi University, 1-1-1 Minami-kogushi, 755-8505 Ube, Yamaguchi, Japan; 2 Department of Internal Medicine, Medical Corporation WADOKAI Hofu Rehabilitation Hospital, Hofu, Japan; 3 Department of Respiratory Medicine, National Hospital Organization Yamaguchi Ube Medical Center, Ube, Japan; 4 Department of Respiratory Medicine and Infectious Disease, Graduate School of Medicine, Yamaguchi University, Ube, Japan; 5 The Department of Clinical Research, National Hospital Organization Yamaguchi Ube Medical Center, Ube, Japan

**Keywords:** Humoral immune response, Immunogenicity, Eastern Cooperative Oncology Group Performance Status Scale, Hypoalbuminemia, Neutralizing activity, Variants of concern, Long-term care facility, Delta variant, Omicron variant

## Abstract

**Background:**

Coronavirus disease 2019 (COVID-19) remains a threat to vulnerable populations such as long-term care facility (LTCF) residents, who are often older, severely frail, and have multiple comorbidities. Although associations have been investigated between COVID-19 mRNA vaccine immunogenicity, durability, and response to booster vaccination and chronological age, data on the association of clinical factors such as performance status, nutritional status, and underlying comorbidities other than chronological age are limited. Here, we evaluated the anti-spike IgG level and neutralizing activity against the wild-type virus and Delta and Omicron variants in the sera of LTCF residents, outpatients, and healthcare workers before the primary vaccination; at 8, 12, and 24 weeks after the primary vaccination; and approximately 3 months after the booster vaccination. This 48-week prospective longitudinal study was registered in the UMIN Clinical Trials Registry (Trial ID: UMIN000043558).

**Results:**

Of 114 infection-naïve participants (64 LTCF residents, 29 outpatients, and 21 healthcare workers), LTCF residents had substantially lower anti-spike IgG levels and neutralizing activity against the wild-type virus and Delta variant than outpatients and healthcare workers over 24 weeks after the primary vaccination. In LTCF residents, booster vaccination elicited neutralizing activity against the wild-type virus and Delta variant comparable to that in outpatients, whereas neutralizing activity against the Omicron variant was comparable to that in outpatients and healthcare workers. Multiple regression analyses showed that age was negatively correlated with anti-spike IgG levels and neutralizing activity against the wild-type virus and Delta variant after the primary vaccination. However, multivariate regression analysis revealed that poor performance status and hypoalbuminemia were more strongly associated with a lower humoral immune response than age, number of comorbidities, or sex after primary vaccination. Booster vaccination counteracted the negative effects of poor performance status and hypoalbuminemia on the humoral immune response.

**Conclusions:**

LTCF residents exhibited suboptimal immune responses following primary vaccination. Although older age is significantly associated with a lower humoral immune response, poor performance status and hypoalbuminemia are more strongly associated with a lower humoral immune response after primary vaccination. Thus, booster vaccination is beneficial for older adults, especially those with a poor performance status and hypoalbuminemia.

## Background

In early 2020, the severe acute respiratory syndrome coronavirus 2 (SARS-CoV-2) infection led to coronavirus disease 2019 (COVID-19), which later disseminated worldwide and claimed numerous lives. With the progress in COVID-19 vaccination and the replacement with the Omicron variant, the mortality and morbidity of COVID-19 have substantially decreased [1]. However, despite the progress in COVID-19 vaccination, older adults and those with multiple comorbidities still have higher levels of mortality and morbidity from COVID-19 than from influenza [2, 3]. Therefore, COVID-19 remains a significant threat to vulnerable populations that are older, severely frail, poorly nourished, and have multiple comorbidities. Although data on immunogenicity suggest that sera from individuals who received booster doses had better neutralizing activity against the Omicron variant [4–7], data on the extent of improvement in older and more vulnerable individuals following booster vaccination compared to that in the general population remains limited.

To develop strategies to protect older and vulnerable populations against the development of severe COVID-19, there is a need to investigate, especially in individuals with the greatest risk of severe COVID-19, the immunogenicity and durability of COVID-19 vaccines, the degree of immune escape by SARS-CoV-2 variants of concern (VOCs) and the booster vaccination effect against that. Although studies have investigated the association between COVID-19 mRNA vaccine immunogenicity, durability, and chronological age [8–12], data on the association between immunogenicity, durability, and clinical factors such as performance status, nutritional status, and underlying comorbidities other than chronological age are limited. Accordingly, better longitudinal evidence on vaccine immunogenicity, durability of immunity, and degree of immune escape by SARS-CoV-2 VOCs, specifically in older and vulnerable individuals, such as residents of long-term care facilities (LTCFs), is required to strategize best practices for controlling infection, preventing outbreaks, and identifying potential indications for further booster vaccinations.

Immune function generally declines with age [13–17], but how and to what extent the humoral immune response to stimulation in vivo changes with clinical factors, such as performance status and nutritional status, other than chronological age remains unknown. In this study, we evaluated this aspect by taking advantage of this rare opportunity for vaccination, in which humans are exposed to uniform antigenic stimulation. In this rare prospective longitudinal 48-week study, not only the kinetics of anti-spike IgG levels and neutralizing activity against the wild-type virus were evaluated, but also neutralizing activity against the Delta and Omicron VOCs of SARS-CoV-2 were determined before and following COVID-19 mRNA primary and booster vaccination in LTCF residents, outpatients, and healthcare workers. Furthermore, we investigated the association between the changes in neutralizing activity with viral mutations and various clinical factors.

Our results could be useful for the development of robust booster strategies as a control measure for SARS-CoV-2 VOCs in older and more vulnerable individuals such as LTCF residents.

## Methods

### Study design and population

Written informed consent was obtained from all participants or their legal guardians. The study protocol adhered to the Declaration of Helsinki and was approved by the Institutional Review Board of Yamaguchi University Hospital (Registration No. 2020–214). This prospective longitudinal study was registered in the UMIN Clinical Trials Registry (UMIN Trial ID: UMIN000043558). The detailed protocol of this study is available at https://center6.umin.ac.jp/cgi-open-bin/ctr/ctr_view.cgi?recptno=R000049712. Other objectives of this study were to: 1) evaluate cellular immunity after COVID-19 vaccination and 2) investigate the relationship between the microbiomes in the intestinal tract and the immunogenicity and durability of the COVID-19 vaccine. However, it takes some time to obtain these results. Therefore, in this paper, we report the results of the humoral immune responses. This study was conducted from March 5, 2021 to July 6, 2022. LTCF residents, outpatients, and healthcare workers were enrolled in this prospective, longitudinal cohort study. The LTCFs included four nursing homes and one long-term care hospital in Yamaguchi, Japan. The outpatients included individuals who regularly visited Yamaguchi University Hospital or Hofu Rehabilitation Hospital in Yamaguchi, Japan. The participants were recruited before they received the COVID-19 vaccine. The eligibility criteria included the absence of SARS-CoV-2 infection before receiving the first vaccine dose.

All participants were asked to provide peripheral blood samples for serological assays at five time points before and during the 48-week period after receiving the first vaccine dose: during the baseline period (before receiving the first vaccine dose), 8 weeks after the first dose (period 1), 12 weeks after the first dose (period 2), 24 weeks after the first dose (period 3), and 48 weeks after the first dose (period 4).

The end of the study for any participant was defined as 350 days after administration of the first vaccine dose, death, or lack of follow-up. A nucleic acid amplification test for SARS-CoV-2 was performed if any COVID-19–associated symptom or exposure to a SARS-CoV-2–infected person was reported. All participants were tested for antibodies specific to the viral nucleocapsid protein (IgG(N)) to rule out a COVID-19 breakthrough infection during the study period (at the baseline period and periods 1, 2, 3, and 4). Individuals with positive results were excluded from the final analysis.

### Serological assays

Serological testing for antibodies to the receptor-binding domain (RBD) of the S1 subunit of the viral spike protein [IgG(S-RBD)] and IgG(N) was performed using the Abbott Architect SARS-CoV-2 IgG II Quant assay and SARS-CoV-2 IgG assay (both Abbott Laboratories, Sligo, Ireland), respectively, according to the manufacturer’s instructions. An IgG(N) S/C ≥ 1.4 denoted seropositive status due to prior infection or SARS-CoV-2 exposure during the observation period, based on a previously established cut-off point [18].

### Surrogate virus neutralization test

A commercially available surrogate virus neutralization test (sVNT; cPass SARS-CoV-2 Neutralization Antibody Detection Kit, Genscript Biotech Corporation, Piscataway, NJ, USA) was used. The surrogate virus neutralization assay had high sensitivity and specificity (with a recommended positive threshold of 30%) and showed an excellent correlation with the plaque reduction neutralization test. The assay detects functional antibodies that neutralize the interaction between the spike protein RBD (spike-RBD) and human angiotensin-converting enzyme 2 (ACE2) [19–21]. The assay was performed in accordance with the manufacturer's instructions. For the Delta and Omicron variant sVNT, the same protocol was followed by replacing the wild-type horseradish peroxidase-conjugated recombinant spike protein RBD (HRP-RBD) with the commercially available recombinant proteins for the Delta (B.1.617.2) and Omicron variants (B.1.1.529, sublineage BA.1) HRP-RBD from Genscript Biotech Corporation.

### Statistical analysis

The data were stratified into three groups: healthcare workers, outpatients, and LTCF residents. Values are summarized as median and interquartile range (IQR) for continuous variables and as frequencies (percentage) for categorical variables. Intergroup differences were tested using Fisher’s exact test for categorical variables and Wilcoxon’s rank-sum test or Kruskal–Wallis test for numerical variables. All pairwise comparisons after the Kruskal–Wallis test were performed using Dunn’s test with Bonferroni correction for multiple testing. Correlations between variables were calculated using Spearman's rank correlation coefficient analysis. Factors of variation of the IgG (S-RBD) level and neutralizing activity against the wild-type virus and the Delta and Omicron variants were analyzed using multiple regression analyses (MRA) that were performed separately for each type of serological assay in periods 1, 3, and 4. As the IgG (S-RBD) levels showed a highly skewed distribution, they were logarithmically transformed before analysis. The optimal regression model was built by a repeated stepwise selection procedure based on the level of adjusted coefficient of determinations. In the selection process, "age" was always included in the model as a control variable to avoid its confounding influence on other parameters. The practical significance of the parameters retained in the regression model was interpreted based on a standardized partial regression coefficient, which corresponds to the partial correlation coefficient (*r*_*p*_) and has values between -1.0 and 1.0. In reference to Cohen's criterion for the effect size of the correlation coefficient [22], we regarded 0.20≦|*r*_*p*_|< 0.3 as "weak", 0.30≦|*r*_*p*_|< 0.5 as "moderate", and 0.5≦|*r*_*p*_| as "strong" correlation. As a sub-analysis, logistic regression analysis was performed to identify potential risk factors for negative neutralizing activity against the Omicron variant observed at 48 weeks. The levels of association were expressed as unadjusted and adjusted odds ratios (ORs) and 95% confidence intervals (CIs). All statistical analyses were performed using StatFlex for Windows Ver. 7 (Artech Inc., Osaka). Scatter plots and box-and-whisker plots were generated using JMP Pro 16.1.0 (SAS Institute Inc., Cary, NC, USA).

## Results

### Study population and serological assays

**Fig. 1:**
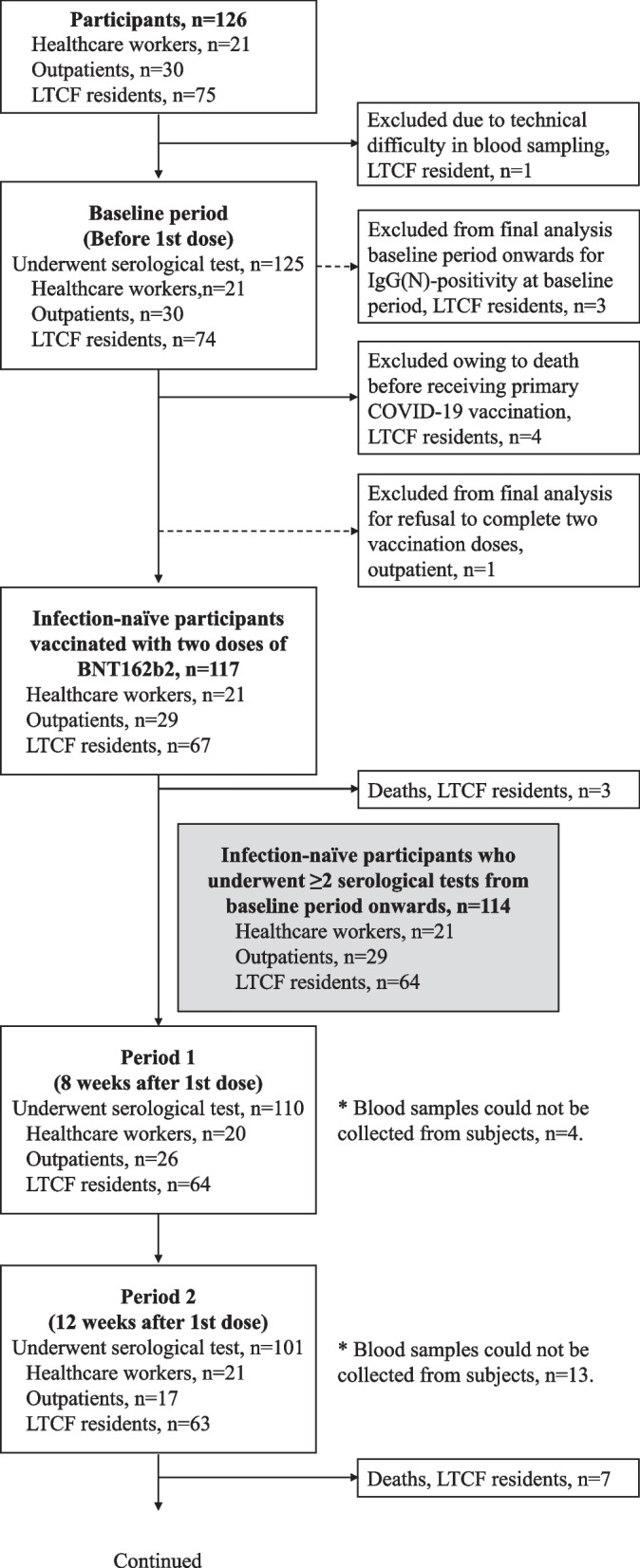

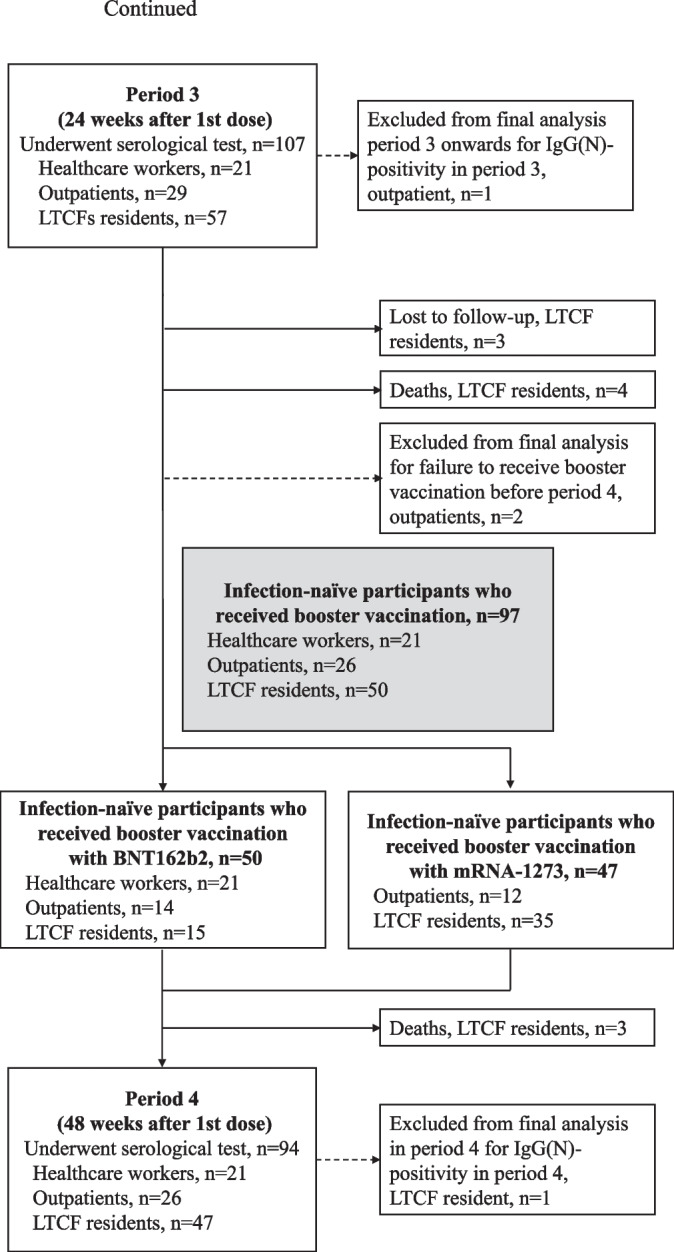
Recruitment of participants, testing, and follow-up. This study included a prospective cohort of residents of long-term care facilities (LTCFs), outpatients, and healthcare workers. During the study period (March 5, 2021, to July 6, 2022), the participants provided peripheral blood samples for serological assays at five time points before and during the 48 weeks after receiving the first vaccine dose: during the baseline period (before the first vaccine dose) and at 8 weeks (period 1), 12 weeks (period 2), 24 weeks (period 3), and 48 weeks (period 4) after the first dose. From the baseline to period 1, one participant refused to complete the two vaccination doses and was excluded from the final analysis. The remaining participants completed two vaccination doses with BNT162b2 (Pfizer-BioNTech) COVID-19 vaccine in the primary vaccine series (two intramuscular doses of 30 mcg each were given three weeks apart) from baseline to period 1. From periods 3 to 4, two participants failed to receive booster vaccination and were excluded from the final analysis in period 4. The remaining participants received booster vaccination from periods 3 to 4. Therefore, the assessment at 48 weeks after the first dose constituted an assessment approximately 3 months after the booster vaccination, wherein all healthcare workers, 14 out of 26 outpatients, and 15 out of 50 LTCF residents received the BNT162b2 (Pfizer-BioNTech) COVID-19 vaccine and 12 out of 26 outpatients and 35 out of 50 LTCF residents received the mRNA-1273 (Moderna) COVID-19 vaccine. Vaccine types for booster vaccination for healthcare workers and LTCF residents were specified by local governments

The final study sample comprised 114 infection-naïve participants (64 LTCF residents, 29 outpatients, and 21 healthcare workers) who underwent at least two serological tests from the baseline period. The sample population consisted of 100% Asian individuals, of whom 60% were females. Detailed baseline demographic characteristics for each subpopulation are summarized in Table 1. The number of participants included in the final analysis who underwent IgG (S-RBD) tests and neutralizing antibody tests at each period is presented in Fig. 1. From the baseline to period 1, one participant refused to complete the two vaccination doses and was excluded from the final analysis. The remaining participants completed two vaccination doses with BNT162b2 (Pfizer-BioNTech) COVID-19 vaccine in the primary vaccine series (two intramuscular doses of 30 mcg each given three weeks apart) from baseline to period 1. From periods 3 to 4, two participants failed to receive the booster vaccination and were excluded from the final analysis in period 4. The remaining participants received booster vaccination from periods 3 to 4. Therefore, the assessment at 48 weeks after the first dose constituted an assessment approximately three months after the booster vaccination, wherein all healthcare workers, 14 out of 26 outpatients, and 15 out of 50 LTCF residents received the BNT162b2 (Pfizer-BioNTech) COVID-19 vaccine, and 12 out of 26 outpatients and 35 out of 50 LTCF residents received the mRNA-1273 (Moderna) COVID-19 vaccine. Vaccine types for booster vaccination for healthcare workers and LTCF residents were specified by the local governments. Although a nucleic acid amplification test for SARS-CoV-2 was performed if any COVID-19–associated symptom or exposure to a SARS-CoV-2–infected person was reported, no COVID-19 patients were identified among the participants during the study period. However, five participants showed positive IgG(N) results and were considered to have been infected with SARS-CoV-2 asymptomatically during the study period; they were excluded from the final analysis.

**Table 1:** Baseline characteristics of the study participants

	Healthcare workers (*N* = 21)		Outpatients (*N* = 29)		Residents of long-term care facilities (*N* = 64)		*P*-value ^a)^
Age, years, (IQR)	51.0	(42.0–60.0)	72.0	(67.0–76.0)	89.5	(84.0–93.5)	< 0.001
Age group, years, n (%)							
< 45	8	(38.1)	0	(0.0)	0	(0.0)	< 0.001
45 to < 65	9	(42.9)	5	(17.2)	2	(3.1)	
65 to < 85	4	(19.0)	23	(79.3)	15	(23.4)	
≥ 85	0	(0.0)	1	(3.5)	47	(73.4)	
Sex, no. (%)							
Male	7	(33.3)	23	(79.3)	16	(25.0)	< 0.001
Female	14	(66.7)	6	(20.7)	48	(75.0)	
Body mass index, (IQR)	21.8	(20.9–23.2)	22.8	(20.7–24.2)	19.4	(17.4–21.1)	< 0.001
ECOG-PS, n (%)							
0	21	(100.0)	22	(75.9)	0	(0.0)	< 0.001
1	0	(0.0)	7	(24.1)	0	(0.0)	
2	0	(0.0)	0	(0.0)	8	(12.5)	
3	0	(0.0)	0	(0.0)	17	(26.6)	
4	0	(0.0)	0	(0.0)	39	(60.9)	
Comorbidity, n (%)							
Chronic respiratory diseases	0	(0.0)	26	(89.7)	10	(15.6)	< 0.001
Chronic heart diseases	0	(0.0)	1	(3.4)	19	(29.7)	< 0.001
Chronic liver diseases	0	(0.0)	2	(6.9)	3	(4.7)	0.493
Chronic kidney disease	2	(9.5)	13	(44.8)	29	(45.3)	0.010
Cerebrovascular diseases	1	(4.8)	3	(10.3)	28	(43.8)	< 0.001
Hypertension	6	(28.6)	14	(48.3)	36	(56.3)	0.088
Diabetes mellitus	1	(4.8)	8	(27.6)	14	(21.9)	0.122
Solid cancer	0	(0.0)	4	(13.8)	5	(7.8)	0.203
Leukemia	0	(0.0)	0	(0.0)	0	(0.0)	NC
Lymphoma	0	(0.0)	0	(0.0)	2	(3.1)	0.451
Acquired immunodeficiency syndrome	0	(0.0)	0	(0.0)	0	(0.0)	NC
Connective tissue diseases	1	(4.8)	1	(3.4)	0	(0.0)	0.256
Immunosuppression ^b)^	1	(4.8)	2	(6.9)	5	(7.8)	0.893
No. of Comorbidities, n (%)							
0	13	(61.9)	0	(0.0)	0	(0.0)	< 0.001
1	5	(23.8)	6	(20.7)	6	(9.4)	
2	3	(14.3)	10	(34.5)	17	(26.6)	
≥ 3	0	(0.0)	13	(44.8)	41	(64.1)	
Functional Independence Measure, (IQR)							
Motor function	91.0	(91.0–91.0)	91.0	(91.0–91.0)	16.5	(13.0–45.0)	< 0.001
Cognitive function	35.0	(35.0–35.0)	35.0	(35.0–35.0)	15.0	(7.0–23.5)	< 0.001
Total score	126.0	(126.0–126.0)	126.0	(126.0–126.0)	30.5	(21.0–64.5)	< 0.001
Mini-mental State Examination, (IQR)	30.0	(30.0–30.0)	27.0	(26.0–30.0)	7.0	(0.0–15.0)	< 0.001
Laboratory data, (IQR)							
Serum total protein level (g/dL)	7.5	(7.2–7.6)	7.0	(6.7–7.3)	6.6	(6.3–7.2)	< 0.001
Serum albumin level (g/dL)	4.6	(4.5–4.8)	4.2	(4.1–4.3)	3.6	(3.3–3.9)	< 0.001
Serum cholesterol (mg/dL)	211.0	(194.0–234.0)	198.0	(171.0–212.0)	165.0	(142.0–190.5)	< 0.001
WBC /mm^3^	5600	(4900–7000)	5670	(4790–6520)	5050	(4045–6645)	0.214
Lymphocyte (%)	32.0	(26.0–36.0)	28.1	(24.2–32.4)	26.1	(19.6–32.0)	0.007
Hemoglobin (g/dL)	14.0	(12.2–14.6)	13.5	(12.8–14.6)	11.1	(10.0–12.7)	< 0.001
Platelet (× 10^4^/μL)	21.0	(17.2–24.6)	20.7	(15.3–24.5)	19.9	(16.1–26.2)	0.873
Total bilirubin (mg/dL)	0.6	(0.5–0.7)	0.7	(0.4–0.8)	0.4	(0.3–0.6)	< 0.001
AST (U/L)	20.0	(18.0–27.0)	23.0	(19.0–31.0)	21.0	(18.5–28.0)	0.357
ALT (U/L)	16.0	(14.0–23.0)	20.0	(16.0–31.0)	15.0	(11.0–22.0)	0.014
LDH (U/L)	168.0	(144.0–180.0)	192.0	(173.0–222.0)	161.0	(141.5–186.5)	< 0.001
e-GFR (mL/min per 1·73 m^2^)	78.6	(68.6–83.7)	61.3	(55.0–75.7)	65.3	(49.7–94.3)	0.071
HbA1c (%)	5.6	(5.5–5.8)	6.0	(5.6–6.4)	5.4	(5.2–5.8)	< 0.001

*Abbreviations:* *IQR* Interquartile range, *ECOG-PS* Eastern Cooperative Oncology Group Performance Status Scale, *WBC* White blood cell count, *AST* Aspartate aminotransferase, *ALT* Alanine transaminase, *LDH* Lactate dehydrogenase, *e-GFR* Estimated glomerular filtration rate, *HbA1c* Glycated hemoglobin, *NC* Not calculable

^a^ Kruskal–Wallis test for continuous variables and Fisher's exact test for categorical variables

^b^ Immunosuppression included being on steroids, immunosuppressive agents, chemotherapy, or biologic therapy

### SARS-CoV-2 IgG (S-RBD) level and neutralizing activity kinetics

**Fig. 2:**
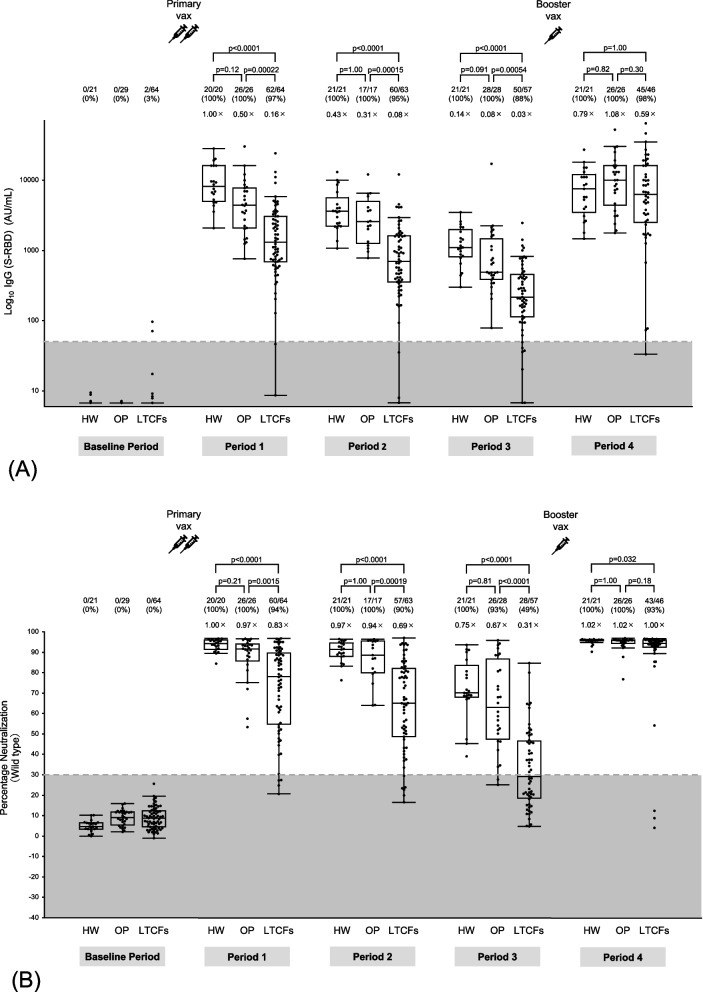

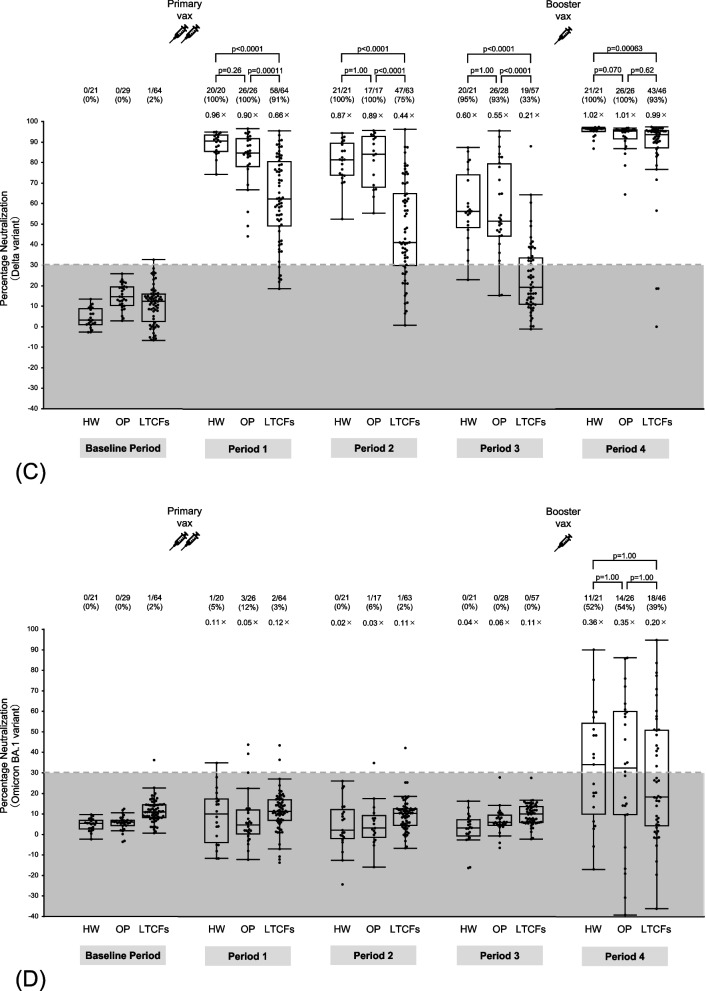
SARS-CoV-2 IgG (S-RBD) and neutralizing activity kinetics. **A** Antibodies to the receptor-binding domain of the S1 subunit of the viral spike protein [IgG (S-RBD)] and neutralizing activity against (**B**) wild-type virus, (**C**) Delta variant, and (**D**) Omicron variants in sera were determined in infection-naïve participants who provided peripheral blood samples for serological assays at five time points during 48 weeks after receiving the first vaccine dose: at baseline (before the first vaccine dose) and at 8 weeks (period 1), 12 weeks (period 2), 24 weeks (period 3), and 48 weeks (period 4) after the first dose. Between 24 (period 3) and 48 weeks (period 4) after the first dose, booster vaccination with BNT162b2 or mRNA-1273 was completed. Period 4 was approximately 3 months after the booster vaccination. The participants were stratified into three subgroups: healthcare workers, outpatients, and residents of long-term care facilities. Each dot represents an individual participant. The boxes span the interquartile range; the line within each box denotes the median, and the whiskers are the largest and smallest values within the range of ± 1.5-fold in the interquartile range from the first and third quartile. The dashed line in panel A indicates a cut-off of 50 AU/mL for assay positivity, whereas those in panels B–D indicate the cut-off of 30% for assay positivity, as determined previously. The gray areas in panels A–D represent negative results. The numbers above each column indicate the number of participants with positive/negative assay results and the proportion of participants with positive assay results. Fold-comparison in geometric mean IgG (S-RBD) levels relative to that in healthcare workers in period 1 is shown as a number with the “ × ” symbol in panel A. Fold-comparison in median neutralizing activity relative to that against the wild-type virus in healthcare workers in period 1 is shown as a number with the “ × ” symbol in panels B–D. *P*-values are indicated above each plot. HW; healthcare workers, OP; outpatients, LTCFs; residents of long-term care facilities

The kinetics of the humoral immune response were assessed throughout the 48-week period (Fig. 2). The sera of LTCF residents had significantly lower IgG (S-RBD) levels and neutralizing activity against the wild-type virus and the Delta variant than those of outpatients and healthcare workers in periods 1–3. During period 3, 51% of LTCF residents showed negative neutralizing activity against the wild-type virus in the sera, whereas only 7% of the outpatients and none of the healthcare workers showed negative results; in this period, 67% of LTCF residents showed negative neutralizing activity against the Delta variant, whereas only 5% of healthcare workers and 7% of outpatients showed negative results. Neutralizing activity against the Omicron variant in the sera was below the cut-off level in most participants throughout the 24 weeks after the first dose, including in healthcare workers during period 1.

In contrast, booster vaccination elicited IgG (S-RBD) levels in LTCF residents comparable to those in healthcare workers and outpatients. Booster vaccination also elicited neutralizing activity against both wild-type virus and the Delta variant in LTCF residents comparable to that in outpatients. Furthermore, the inter-individual differences in neutralizing activity against the wild-type virus and the Delta variant decreased conspicuously after the booster vaccination in all subgroups, with a few exceptions. Meanwhile, in LTCF residents, the booster vaccination elicited neutralizing activity against the Omicron variant comparable to that in healthcare workers and outpatients. However, only 46% of the participants who received booster vaccines exhibited positive neutralizing activity against the Omicron variant, and the inter-individual differences remained high.

### Potential factors responsible for the variation in IgG (S-RBD) levels and neutralizing activity

Factors that are likely involved in the variation in IgG (S-RBD) levels and neutralizing activity against the wild-type virus and the Delta and Omicron variants were analyzed using MRA (Table 2). Age-related changes in IgG (S-RBD) and neutralizing activity were first examined univariately by MRA, and their magnitude was expressed by the standardized partial regression coefficients (*r*_*p*_). By setting |*r*_*p*_|≥ 0.20 as a practical level of importance, age was negatively correlated with IgG (S-RBD) levels, neutralizing activity against the wild-type and Delta variant during periods 1 and 3, and with neutralizing activity against the Delta variant in period 4. However, according to the multivariate analysis, age was not independently associated with the variation of IgG (S-RBD) and neutralizing activity, except for IgG (S-RBD) in period 1 and neutralizing activity against wild-type virus and Delta variant in period 4. Furthermore, serum albumin showed positive correlations with neutralizing activity against the wild-type virus and the Delta variant in period 1 and with the wild-type virus in period 3. Additionally, the Eastern Cooperative Oncology Group Performance Status Scale (ECOG-PS) score showed a negative correlation with IgG (S-RBD) level in periods 1 and 3 and with the neutralizing activity against the wild-type virus and the Delta variant in period 3. MRA was not performed for neutralizing activity against the Omicron variant in periods 1 and 3, because the activity was below the cut-off level in most participants. In period 4, after the booster vaccination, age was negatively correlated with neutralizing activity against the wild-type and Delta variant. Meanwhile, no significant factors were found to be associated with the variation in neutralizing activity against the Omicron variant after booster vaccination, except for minor sex-related differences. Logistic regression analysis was performed as a sub-analysis to identify potential risk factors for negative neutralizing activity against the Omicron variant in period 4 (Table 3). However, no significant factors were identified by either univariate or multivariate analyses.

**Table 2.**
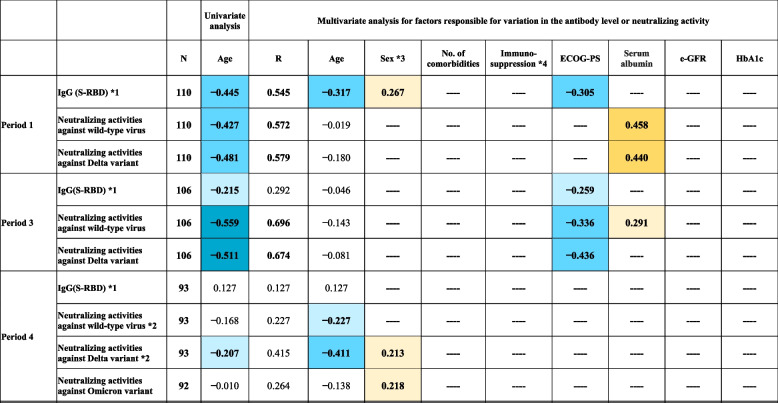
Multiple regression analyses to investigate factors responsible for the variation in IgG (S-RBD) and neutralizing activities against the wild-type virus and the Delta and Omicron variants

The levels of each of the antibodies to the receptor-binding domain (RBD) of the S1 subunit of the viral spike protein [IgG (S-RBD)] and neutralizing activities were set as objective variables, and the following factors were considered as candidate explanatory variables: age, sex, number of comorbidities, immunosuppression (binary), Eastern Cooperative Oncology Group Performance Status Scale (ECOG-PS), serum albumin, estimated glomerular filtration rate (e-GFR), and glycated hemoglobin (HbA1c). The values presented in the table are r_p_ of statistical significance (*P* < 0.05) except for that in the column of n (data size) and R (multiple regression coefficient). Values of R ≥ 0.5 are shown in bold. The values of |r_p_| are shown in three graded background colors: orange if positive and blue if negative. The graded color corresponds to slight (0.2 ≤|r_p_|< 0.3), moderate (0.3 <|r_p_|< 0.5), and strong (0.5 ≤|r_p_|) correlations

^*^1 The antibody level was logarithmically transformed before the analysis

^*^2 The neutralizing activity was transformed into a ranking scale to correct for highly skewed distribution before the analysis

^*^3 The dummy variable "sex" was coded as male = 0 and female = 1

^*^4 Immunosuppression included receiving steroids, immunosuppressive agents, chemotherapy, or biologic therapy

**Table 3:** Logistic regression analysis to investigate the risk factors for negative neutralizing activity against the Omicron variant after booster vaccination

	**Unadjusted**		**Adjusted**	
Characteristics	**OR (95% CI)**	***p*-value**	**OR (95% CI)**	***p*-value**
Age, (years)	1.010 (0.987, 1.034)	0.390	0.996 (0.963, 1.031)	0.831
Sex, female *1	0.903 (0.394, 2.071)	0.809		
Booster vaccination type, mRNA-1273 (Moderna) *2	1.630 (0.716, 3.711)	0.383	1.123 (0.421, 2.994)	0.817
Period from the first dose to booster dose, (days)	0.999 (0.976, 1.023)	0.950		
Period from the booster dose to blood sampling, (days)	1.008 (0.982, 1.035)	0.543		
ECOG-PS	1.168 (0.922, 1.481)	0.198	1.098 (0.744, 1.620)	0.639
No. of comorbidities	1.139 (0.868, 1.495)	0.348		
Immunosuppression	6.837 (0.806, 57.996)	0.078	6.226 (0.705, 54.956)	0.100
Serum albumin level (g/dL)	0.613 (0.261, 1.441)	0.262	0.783 (0.176, 3.489)	0.749
e-GFR (mL/min per 1·73 m^2^)	1.012 (0.994, 1.031)	0.184		
HbA1c (%)	0.803 (0.384, 1.681)	0.560		

*Abbreviations:* *ECOG-PS* Eastern Cooperative Oncology Group Performance Status Scale, e-GFR, Estimated glomerular filtration rate, *HbA1c* Glycated hemoglobin

^*^1 The dummy variable "sex" was coded as male = 0 and female = 1

^*^2 The dummy variable "Booster vaccination type" was coded as BNT162b2 (Pfizer-BioNTech) = 0 and mRNA-1273 (Moderna) = 1

### Correlation between IgG (S-RBD) level and neutralizing activity

**Fig. 3:**
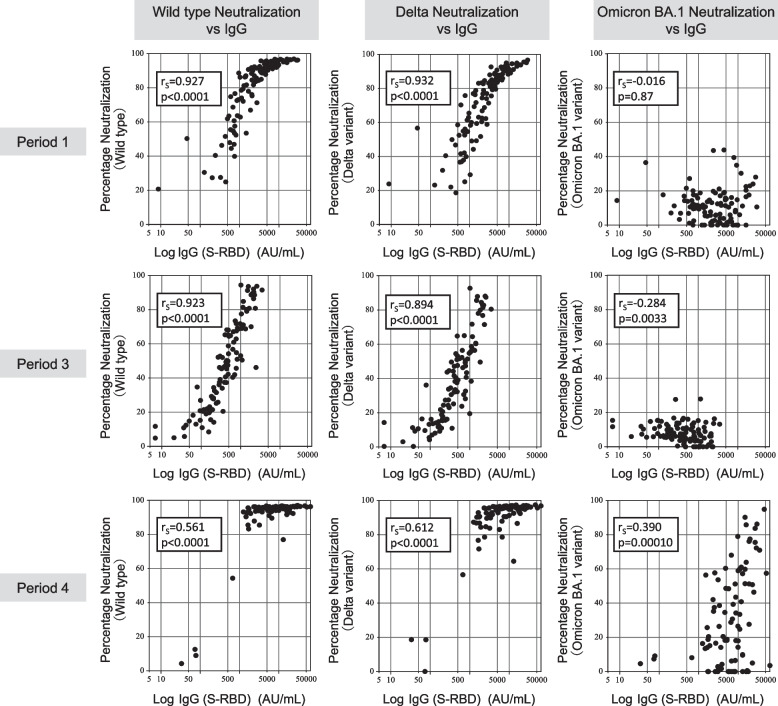
Correlation between IgG (S-RBD) and neutralizing activity. We assessed the correlation between antibodies to the receptor-binding domain of the S1 subunit of the viral spike protein [IgG (S-RBD)] and neutralizing activity against the wild-type virus and the Delta and Omicron variants. The left, middle, and right columns show correlations between neutralizing activity against the wild-type virus and the Delta and Omicron variants, respectively, and the IgG (S-RBD) levels. The upper, middle, and lower rows show the correlations in periods 1, 3, and 4, respectively. The neutralizing activity was plotted as zero when the recordings were negative

We assessed the correlation between the IgG (S-RBD) level and neutralizing activity against the wild-type virus and the Delta and Omicron variants (Fig. 3). IgG (S-RBD) level and the neutralizing activity against the wild-type virus and the Delta variant showed a strong positive correlation in periods 1, 3, and 4 (Spearman’s rank correlation: 0.561–0.932). Meanwhile, no significant correlation was found between the IgG (S-RBD) level and neutralizing activity against the Omicron variant in period 1 (Spearman’s rank correlation: -0.061). Although IgG (S-RBD) level and the neutralizing activity against the Omicron variant appeared to show a weak negative correlation in period 3 (Spearman’s rank correlation: -0.284), this was regarded as irrelevant as the neutralizing activity against the Omicron variant was below the cut-off level in most participants. In contrast, in period 4, IgG (S-RBD) levels and neutralizing activity against the Omicron variant showed an appreciable positive correlation (Spearman’s rank correlation: 0.390).

## Discussion

The aging immune system undergoes immune-senescence, which can result in impaired vaccine responses [13]. Immune-senescence-related changes include reduced immune function, such as constrained germinal center responses, a reduced naive cell repertoire, accumulation of an expanded memory pool, and an increase in inflammatory subsets of adaptive immune cells [14–17]. In fact, the negative impact of age on immunogenicity with COVID-19 vaccination was highlighted recently [23–31]. In the present study, LTCF residents exhibited suboptimal immune responses following primary vaccination. These results were consistent with previous reports, indicating a lower intensity of humoral immune response and a narrower breadth of cross-neutralization in older and more vulnerable individuals than in the general population.

However, the humoral responses to vaccination showed large inter-individual differences. Results of multivariate regression analysis showed that poor ECOG-PS and hypoalbuminemia were more strongly associated with a lower humoral immune response than age, number of comorbidities, and sex after the primary vaccination series. Although older age was an important factor associated with a lower humoral immune response, vulnerable individuals, particularly those with poor ECOG-PS and hypoalbuminemia, showed a lower humoral immune response. Older adults with frailty show impaired vaccine effectiveness, including influenza, varicella-zoster, and pneumococcal pneumonia vaccine [32–34]. Furthermore, frailty is an independent predictor of impaired antibody responses to COVID-19 mRNA vaccines [35, 36]. Physical inactivity has also been shown to be a risk factor for severe COVID-19 [35, 37]. Moreover, there is a dose–response relationship suggesting that the higher the physical activity, the higher the efficacy of vaccines, including the COVID-19 vaccine [38–42]. These findings suggest that immunogenicity with COVID-19 vaccination could be improved by encouraging exercise in frail and older individuals through appropriate rehabilitation programs. Physical inactivity triggers persistent low-grade systemic inflammation, which may cause immune system dysfunction [43]. Inhibitory substances associated with inflammatory states, such as tumor necrosis factor and interleukin-1, impede albumin synthesis [44–46]. Among the participants in this study, those with hypoalbuminemia might have had latent chronic inflammation, in addition to malnutrition. This may be one of the mechanisms by which poor ECOG-PS and hypoalbuminemia were associated with a lower humoral immune response. Although this study did not aim to clarify these mechanisms, further studies should investigate the mechanisms underlying the low humoral immune response, which might herald the identification of measures to improve immunogenicity in vulnerable populations.

Compared to antibody levels, which decline over time, memory B cells exhibit a more sustained presence following vaccination and/or SARS-CoV-2 infection [47]. Some studies have demonstrated that after COVID-19 mRNA vaccination, B cells continue to undergo affinity maturation [47–50], which facilitates improved antibody functionality in neutralizing the virus. Thus, booster doses can increase the levels of antibodies and enhance the breadth of the immune response against SARS-CoV-2 [4–7]. However, the specific duration of B cell persistence, affinity maturation, and extent of booster effect may vary among individuals and may be influenced by factors such as age, performance status, and nutritional status. In this study, LTCF residents exhibited suboptimal immune responses with primary vaccination series alone. However, booster vaccination elicited an immune response in LTCF residents comparable to that in healthcare workers and outpatients. Of the LTCF residents in the present study, 87.5% had an ECOG-PS score of 3 or higher. Notably, even in LTCF residents with poor ECOG-PS and hypoalbuminemia, booster vaccination elicited humoral immune responses comparable to those in the general population. Furthermore, inter-individual differences in neutralizing activity against the wild-type virus and the Delta variant decreased after the booster vaccination. Notably, after the booster vaccination, the humoral immunity was enhanced relatively uniformly among vulnerable older individuals, outpatients, and healthcare workers. The booster vaccination counteracted the negative effects of poor performance status and hypoalbuminemia on humoral immune responses, as shown in Table 2. Thus, booster vaccination is particularly beneficial for older adults, especially LTCF residents with poor ECOG-PS and hypoalbuminemia.

Although booster vaccination elicited a higher neutralizing activity against the Omicron variant than the primary vaccination series in all subgroups, only 46% participants exhibited positive neutralizing activity against the Omicron variant, even after the booster vaccination, and inter-individual differences remained very high. The risk factors for negative neutralizing activity against the Omicron variant after booster vaccination are unknown. Unlike that observed with the wild-type and Delta variant after the primary vaccination, older vulnerable individuals did not show particularly poor neutralizing activity against the Omicron variant after the booster vaccination, as shown in Tables 2 and 3. There are healthy non-responders to the hepatitis B virus vaccination. The lack of response appears to be genetically determined and related to the human leukocyte antigen haplotypes [51]. Our sample population was limited to a single geographic region in Japan and was 100% Asian. Although a simple comparison is not possible owing to different measurement methods, healthcare workers and outpatients in the present study appeared to show a lower acquisition rate for neutralizing antibodies against the Omicron variant after the booster vaccination in comparison to previous reports from the United States and Israel [4, 5, 7]. However, whether there are any genetic basis or racial differences in the non-development of neutralizing activity against the Omicron variant following the COVID-19 booster vaccination based on the wild-type virus remains unclear and further research is required to clarify this issue.

Herein, IgG (S-RBD) levels and neutralizing activity against the wild-type virus and the Delta variant showed a positive correlation. Since the presence of neutralizing antibodies is indicative of protection [52, 53], our observations suggest that the results of IgG (S-RBD) testing may be used to predict protection from the wild-type virus and Delta variant as an alternative test to VNT. In contrast, no significant correlation was found between the IgG (S-RBD) level and neutralizing activity against the Omicron variant after the primary vaccination. Therefore, when Omicron was the dominant strain worldwide, protection from infection could not have been predicted based on the IgG test results. Nonetheless, booster vaccination elicited a better correlation between IgG (S-RBD) levels and neutralizing activity against the Omicron variant than the primary vaccination series, highlighting the notable advantage of booster vaccination. Moreover, the data in Fig. 3 show that the neutralizing activity was enhanced after the booster dose, even at similar IgG (S-RBD) levels. In particular, the phenomenon was observed at IgG (S-RBD) levels within the range of 500–5000 AU/mL for the wild-type virus and the Delta variant and at IgG (S-RBD) levels > 5000 AU/mL for the Omicron variant. These findings suggest the persistence of SARS-CoV-2-specific B cells that continue to undergo affinity maturation even in older vulnerable individuals. However, even after booster vaccination, the correlation between IgG (S-RBD) levels and neutralizing activity against the Omicron variant was weak. Most previous studies evaluating immunogenicity after COVID-19 vaccination in LTCF residents only evaluated IgG antibody levels. Therefore, the results of this study, which evaluated the neutralization activity against VOCs, including the Omicron variant, are valuable in considering infection control measures for LTCF residents.

The main limitation of our study is the small sample size, owing to which it is difficult to firmly establish the effects of performance status and hypoalbuminemia. Whether other antibody-mediated functions, such as complement deposition, antibody-dependent cellular cytotoxicity, and antibody-dependent cellular phagocytosis, are lower in older vulnerable individuals than in the general population remains an important but unresolved issue. Moreover, it remains unclear whether there are differences in the long-term durability of the humoral immune response after booster vaccination between vulnerable older individuals and the general population. Further research is needed to answer these questions. These findings will enable the development of robust boosting strategies as control measures for SARS-CoV-2 VOCs in older vulnerable individuals.

## Data Availability

The data are available to approved individuals upon reasonable request to the Yamaguchi University after fulfilling specific requirements.
